# Traditional growing rod for early-onset scoliosis in high-altitude regions: a retrospective study

**DOI:** 10.1186/s13018-021-02639-4

**Published:** 2021-08-10

**Authors:** Haijun Jiang, Junrui Jonathan Hai, Peng Yin, Qingjun Su, Shiqi Zhu, Aixing Pan, Yunsheng Wang, Yong Hai

**Affiliations:** 1 Department of Orthopedic Surgery, Liangxiang Teaching Hospital, Capital Medical University, Beijing, 102401 China; 2 High School Affiliated to Renmin University of China, Beijing, China; 3 Department of Orthopedic Surgery, Beijing Chao-yang Hospital, Capital Medical University, No. 8 Gongren Tiyuguan Nanlu, Chaoyang District, Beijing, 100020 China

**Keywords:** Early-onset scoliosis, High altitude, Traditional growing rod, Complications, Deformity

## Abstract

**Background:**

Children with early-onset scoliosis living in high-altitude areas have severe deformities and poor nutritional status. However, no reports on early-onset scoliosis treatment using traditional growing rods in such children exist. Thus, we analyzed the outcomes of traditional growing rods treatment in such patients and the effect of altitude on therapy.

**Methods:**

Between September 2007 and December 2017, 59 consecutive patients with EOS underwent systematic surgical correction using traditional growing rods. They were divided into the high-altitude and low-altitude groups, and differences in surgical efficacy and complications between the groups were analyzed pre- and postoperatively. Radiographic measurements, including the Cobb angle, thoracic kyphosis, lumbar lordosis, T1–S1 and T1–T12 heights, sagittal and coronal balance, distance between C7PL and sagittal vertical axis, pelvic incidence, sacral slope, and pelvic tilt were assessed preoperatively, postoperatively, and at the last follow-up. Continuous data were analyzed using paired or independent Student’s *t* tests, and they were compared preoperatively, postoperatively, and at the last follow-up using a repeated measures analysis of variance. Enumerated data were analyzed using the *χ*^2^ test.

**Results:**

The mean patient age at the initial surgery and mean follow-up duration were 8.9 ± 2.4(5–14) years and 51.91 ± 25.23 months, respectively. Altogether, 234 operations were conducted for all patients with an average interval between operations of 11.4 ± 3.0 months. The average Cobb angle was similar in both groups preoperatively and at the last follow-up, it was significantly different postoperatively. TK was significantly different in all three periods. T1–S1 and T1–T12 heights were significantly different only during the preoperative period. The overall rates of complications and implant-related complications did not differ significantly between the groups.

**Conclusions:**

Deformity in patients with EOS in high-altitude areas was more severe, and treatment using TGRs yielded a satisfactory therapeutic effect.

## Introduction

Early-onset scoliosis (EOS) is defined as scoliosis with an onset before the age of 10 years. Its etiology and clinical manifestations are diverse and complex [1]. EOS progresses rapidly, leading to severe and complex deformities, which not only affect the longitudinal growth of the child, causing shortness of the spine and trunk, but also cause severe lung function damage [2, 3]. Lung development is not completed until approximately 8 years of age; thoracic deformity seriously affects lung maturity, and thus, it is often accompanied by pulmonary dysfunction [4]. Conservative treatments are mainly ineffective, and scoliosis progression with a coronal Cobb angle > 45° should be treated surgically. The principle of surgical treatment is to correct scoliosis and maintain the corrective effect during the subsequent growth period, preserve spinal growth capacity, maintain normal lung and thorax development, avoid premature terminal orthopedic fusion surgery, and minimize neurological complications [5].

Traditional growing rods (TGRs) are one of the non-fusion scoliosis correction surgeries that preserve spinal growth capacity. For decades, this technology has been used by many spine surgeons to treat EOS [3, 5]. The coronal correction rate and T1–S1 growth height of patients treated with a dual growing rod were better than those in patients treated with a single growing rod. Additionally, the complication rate of the single growing rod group was higher than that of the dual growing rod group, although the dual growing rod group had more wound complications [6]. Magnetically controlled growing rods (MCGRs) can achieve the same correction rate of coronal deformity as the TGR, although coronal deformity and spinal rotation deformity are only improved significantly at the first implantation. Moreover, there is no significant improvement in the subsequent expansion process [7]. The vertical expandable titanium rib prosthesis can stabilize the chest wall segment and expand to the outside but can achieve little deformity correction; thus, complications and reoperation rates remain very high [8]. SHILLA technology cannot reduce the incidence of complications and reoperation rates [9]. Growing rods can provide good spinal control, the dual growing rod has stronger biomechanics and lowers implant-related complications, and less soft tissue coverage may lead to incision complications [6]. Therefore, there is no perfect surgical technique to better treat early-onset scoliosis. Under the guidance of the concept of translational medicine, 3D printing technology has been applied in the field of orthopaedics. 3D simulation becomes an effective tool in order to help surgeons to understand many complex problems. In recent years, our institute has begun to use 3D printing for preoperative evaluation of surgical treatment of scoliosis [10].

People living in high-altitude areas for a long time are generally shorter in height and lighter and have different forms of thoracic structures, which may be related to malnutrition, poor medical conditions, and chronic hypoxia than people living in low-altitude areas [11]. Over 50% of children in Tibet, China, have moderate to severe stunting, and the proportion of severely stunted children in non-urban areas is higher than that in urban areas. Thus, high altitude may cause stunted growth in Tibetan children, which is not related to socioeconomic factors such as nutrition and disease; with an increase in altitude, the incidence of stunting increased significantly, and a significant positive correlation was observed between the prevalence of underweight and altitude. Moreover, the risk of stunting in children living in areas over 3500 m was 2–6 times higher than that of children living in areas over 3000 m [12]. Hypoxia in high-altitude areas aggravates the occurrence of various deformities of the vertebral body in patients with congenital scoliosis, and the proportion of rib deformities may be higher and more severe, affecting the development of the spinal cord [13].

EOS causes great harm to the growth and development of children. Children in high-altitude areas have poor nutritional status and live in hypoxic environments for a long time, and the proportion of stunted growth among them is very high. Congenital scoliosis is common in children living at high altitudes and is characterized by more severe rib deformities and intra-spinal malformations [12]. Currently, there are no reports on the treatment of EOS using growing rods in children in high-altitude areas. Due to the support of the rescue fund, the proportion of patients with EOS from high-altitude areas is higher in our department. This study aimed to investigate the effects and complications of growing rod use for treating EOS in children in high-altitude areas.

## Methods

### Patient selection

This study retrospectively analyzed 62 children with EOS who received TGR treatment at Beijing Chao-yang Hospital between September 2007 and December 2017. The inclusion criteria in our study were as follows: diagnosis of EOS and treatment with TGRs, follow-up for over 2 years, lengthening surgery performed more than once, and rod adjustment surgery with an interval of 6–12 months. Studies with lacking follow-up time, incomplete data, or vague influence were excluded. Measurements were performed independently by two of the authors using a picture archiving and communication system (PACS). All the radiographs were calibrated to achieve accurate distance measurements. Clinical demographic data including age at the initial operation, sex, height, weight, body mass index, and diagnosis (idiopathic, neuromuscular, congenital, or syndromic). Living altitude was evaluated. Surgical information including single or dual growing-rod surgery, instrumented segments, fixation type (pedicle screw, hook, or hybrid), submuscular or intramuscular approach, and the number of lengthening surgeries were analyzed, and low-altitude geographic regions were defined as altitudes≤ 500 males and high-altitude geographic regions as altitudes ≥ 3000 males in our study. The patients were divided into the high-altitude group (H-A group) and low-altitude group (L-A group), and the differences in surgical efficacy and complications between both groups pre- and postoperatively were analyzed. The study protocol was reviewed and approved by the Committee on Ethics and the institutional review board of Beijing Chao-Yang Hospital, Capital Medical University. All methods were performed in accordance with the relevant guidelines and regulations, and a consent form was signed by parents of each child.

### Radiographic evaluation

PACS was used for radiographic measurements preoperatively, postoperatively, and at the last follow-up with growing rods. Posteroanterior and lateral radiographs of the entire spine were taken in all patients. AP radiographic measurements included the Cobb angle, thoracic kyphosis (TK), lumbar lordosis (LL), T1–S1 range (mm), T1–T12 range (mm), sagittal balance, coronal balance, distance between the C7PL and sagittal vertical axis, pelvic incidence (PI), sacral slope (SS), and pelvic tilt (PT).

### Surgical technique

All operations were performed under general anesthesia. During the operation, Neurophysiological monitoring was performed. The skin incision was a midline long incision or two incisions at the upper and lower ends. Patients with unbalanced trunks were treated with dual rod fixation, while those with well-balanced trunks or those who were thin were treated with single rod fixation. The stable vertebrae and the skull to the upper thoracic curve were selected as the lower instrumental vertebra (LIV) and the upper instrumental vertebra (UIV), respectively. The upper and lower fixation areas were stripped under the periosteum to protect the integrity of the joint capsule. Pedicle screws can increase the structural stability. Hook or hybrid fixations can also be used. The distal end can be fixed with pedicle screws, and the distal and proximal anchor points can be fused with limited fusion. To increase the stability, the connecting rod should be bent for consistency with the curvature of the physiological kyphosis and linked with the connector, and proper expansion should be carried out. The extension time was determined according to age, diagnosis, sitting height, and curve progression forgenerally 6–12 months, and final correction was suggested for patients who had well-developed lung function and bones, a Risser sign > 1°, or menstruation in female patients.

### Statistical analysis

The analyses were performed using SPSS (version 24.0; IBM, Armonk, NY, USA), and descriptive statistics were expressed as numbers and percentages for categorical variables. Paired or independent Student’s *t* tests were used to analyze continuous data. The *χ*^2^ test was used to analyze enumerated data. A repeated measures analysis of variance was used to compare continuous data preoperatively, postoperatively, and at the last follow-up within groups.

## Results

### General characteristics

Altogether, 59 patients from our department treated between 2007 and 2017 met the inclusion criteria, with 23 in the H-A group (10 boys,13 girls) and 36 in the L-A groups(14 boys, 22 girls). The baseline data are shown in Table 1. In terms of etiology, scoliosis was idiopathic in 17 patients (7 patients in the H-A group; 10 in the L-A group), congenital in 34 patients (13 patients in the H-A group; 21 in the L-A group), neuromuscular in 4 patients (1 patient in the H-A group; 3 in the L-A group), and neuromuscular in 4 patients (2 patients in each group). Age at the initial growing-rod implantation was not significantly different, although weight, height, and BMI were lower in the H-A group than in the L-A group (*P* < 0.05). The average follow-up was longer in the H-A group than in the L-A group, although it was not significantly different. Radiographs of 234 surgeries were available for measurement (89 in the H-A group, 145 in the L-A group), and an average of 3.97 lengthening procedures per patient was performed (3.87 in the H-A group, 4.03 in the L-A group). Altogether, 31 patients (9 in the H-A group; 22 in the L-A group) underwent definitive fusion follow-up. Single rods were used in 36 surgeries (6 in the H-A group; 30 in the L-A group), while dual rods were used in 23 surgeries (17 in the H-A group; 6 in the L-A group). This difference was significant in both groups (*P* < 0.05). The UIV and LIV distributions are listed in Table 2. The distal and proximal fixations are shown in Table 3.

**Table 1:** Baseline data

Index	H-A groups	L-A groups	Total	*P* value
No. of patients	23	36	59	0.492
Sex (male/female)(no.)	10/13	14/22	24/35	0.726
Age at initial growing-rod implantation (year)	8.17 ± 2.12 (5–14)	9.36 ± 2.54 (5–14)	8.9 ± 2.4 (5–14)	0.064
Height (cm)	110.39±11.14 (94–140)	122.35±12.29 (95–141)	94–141 (117.68 ± 13.14)	0.00
Weight (kg)	19.39 ± 5.05 (11–34)	26.49±7.93 (13–45)	23.72 ± 7.73 (11–45)	0.00
BMI	15.40 ± 2.15 (11.54–19.45)	17.32 ± 3.10 (13.08–23.92)	16.57 ± 2.91 (11.54–23.92)	0.036
Diagnosis				0.291
Idiopathic	9	21	31	
Congenital	10	8	18	
Neuromuscular	4	6	10	
Syndromic	0	1	1	
Duration of follow-up (months)	54.72 ± 26.42 (24–111)	45.65 ± 22.71 (24–132)	51.91 ± 25.23 (24–132)	0.18
Surgical procedures per patient (no.)	89/3.87	145/4.03	234/3.97	
Patients with final fusion (no.)	9	22	31	0.099
Single/dual rods (no.)	6/17	30/6	36/23	0.00
Subcutaneous/submuscular (no.)	12/11	18/18	30/29	0.871
Number of surgical procedures	3.87 ± 1.36 (2–7)	4.03 ± 1.93 (2–10)	3.97 ± 1.72 (2–10)	0.732
The average interval of operations (months)	12.17 ± 2.59 (5–24)	10.94 ± 3.15 (4–24)	11.4 ± 3.0 (4–24)	0.08

**Table 2:** Distribution of UIV and LIV

	H-A group	L-A group	Total
UIV			
C6	0	1	1
C7	0	1	1
T1	2	5	7
T2	16	15	31
T3	4	8	12
T4	0	5	5
T5	0	1	1
T8	0	1	1
LIV			
T12	0	1	1
L1	2	1	3
L2	3	1	4
L3	7	15	22
L4	9	9	18
L5	2	9	11

**Table 3:** Distal and proximal fixation in the two groups

	H-A group	L-A group	Total
UIV			
Screw	8	12	20
Hook	0	5	5
Hybrid	15	19	34
LIV			
Screw	23	35	58
Hook	0	0	0
Hybrid	0	1	1

### Radiological parameters

The average Cobb angle was similar in both groups preoperatively and at the last follow-up (*P* > 0.05), although the difference was significant (*P* < 0.05). TK was different preoperatively, postoperatively, and at the last follow-up (*P* < 0.05). T1–S1 height and T1–T12 height were different preoperatively (*P* < 0.05), although they were not different postoperatively and at the last follow-up (*P* > 0.05).

The major curve Cobb angle increased from a median of 87.8° (range 47.1–126.2°) preoperatively to 48.8° (range 5.1°–73.5°) at the last follow-up (*P* < 0.05) in the H-A group. The major curve Cobb angle decreased from a median of 98.3° (range 52.9°–130.5°) preoperatively to 47.56° (range 17.5°–81.3°) at the last follow-up (*P* < 0.05) in the L-A group. The major TK angle decreased from a median of 81.37°(range 6°–145.4°) preoperatively to 50.1° (range 16.5°–81.5°) at the last follow-up (*P* < 0.05) in the H-A group. The median TK angle decreased from 54.14° (range 4.4°–102.8°) preoperatively to 38° (range 9.7°–82.5°) at the last follow-up (*P* < 0.05) in the L-A group; medianT1–S1 distance increased from a median of 228.82 mm (range 160.41–318.06 mm) preoperatively to 341.25 mm (range 263.93–464.87 mm) at the last follow-up (*P* < 0.05) in the H-A group. Median T1–S1 distance increased from a median of 260.88 mm (range 154.68–359.75 mm) preoperatively to 367.08 mm (range 254.93–465.51 mm) at the last follow-up (*P* < 0.05) in the L-A group. Median T1–T12 height increased from a median of 128.26 mm (range 68.1–190.48 mm) preoperatively to 204.82 mm (range 144.8–259.06 mm) at the last follow-up (*P* < 0.05) in the H-A group. Median T1–T12 height increased from a median of 153.34 mm (range 69.3–224.6 mm) preoperatively to 216.88 mm (range 123.35–292.06 mm) at the last follow-up (*P* < 0.05) in the L-A group. For LL, it was similar in the L-A group (*P* = 0.213) preoperatively and at the last follow-up, while it significantly decreased from the preoperative period to the last follow-up (*P* < 0.05) in the H-A group. C7PL–CSVL was similar in the H-A group (*P* > 0.05) preoperatively and at the last follow-up. C7PL–CSVL was significantly corrected from the preoperative period to the last follow-up (*P* < 0.05). C7PL–CSVL, PI, PT, and SS were all similar between both groups (*P* > 0.05) preoperatively and at the last follow-up (Table 4). Radiological results of all patient groups are shown in Table 5.

**Table 4:** Pelvic parameters in all patients and between the two groups

Index	H-A group	L-A group	Total	P value
Pelvic incidence				
Preoperation (°)	38 ± 12.45 (16–64)	40.34 ± 10.65 (19.2–58.8)	39.43 ± 11.24 (16–64)	0.786
Postoperation (°)	43.25 ± 8.86 (21.7–62)	38.41 ± 11.92 (16.3–60)	40.29 ± 11.01 (16.3–62)	0.459
Last follow-up (°)	40.05 ± 8.87 (19.3–53.4)	39.88 ± 11.85 (15.2–64.3)	39.95 ± 10.71 (15.2–64.3)	0.476
Pelvic tilt				
Preoperation (°)	5.83 ± 11.71 (− 18–11.7)	− 9.2 ± 46.4 (8.86–11.31)	7.68 ± 11.46 (− 18–46.4)	0.786
Postoperation (°)	9.36 ± 9.26 (−10.3–28.3)	7.03 ± 13.02 (−10.3–28.3)	7.94 ± 11.67 (−22.6–28.3)	0.459
Last follow-up (°)	4.43 ± 11.2 (− 13.5–37)	6.37 ± 9.56 (− 15–30.5)	5.62 ± 10.1(− 15–37)	0.476
Sacral slope				
Preoperation (°)	32.17 ± 9.26 (12.7–46.1)	32.79 ± 5.64 (20–42.6)	32.54 ± 7.2 (12.7–46.1)	0.750
Postoperation (°)	33.89 ± 6.37 (18–48)	31.37 ± 8.81 (10–46.4)	32.35 ± 7.99 (10–48)	0.24
Last follow-up (°)	35.62 ± 7.29 (12.4–47.2)	33.51 ± 7.82 (18.2–50)	34.33 ± 7.62 (12.4–50)	0.302
Sagittal balance				
Preoperation (mm)	23.09 ± 19.35 (0–60.61)	34.99 ± 26.39 (0–103.26)	30.35 ± 24.42 (0–103.26)	0.09
Postoperation (mm)	34.26 ± 26.54 (4.67–95.29)	31.61 ± 23.62 (4.15–107.91)	30.35 ± 24.42 (4.15–107.91)	0.926
Last follow-up (mm)	35.72 ± 23.11 (4.19–91.09)	35.69 ± 28.97 (0–109.21)	35.70 ± 26.63 (0–109.21)	0.997
Coronal balance				
Preoperation (mm)	19.27 ± 18.49 (4.09–73.76)	34.8 ± 27.7 (0–102.76)	28.75 ± 25.52 (0–102.76)	0.019
Postoperation (mm)	27.29 ± 26.06 (4.09–73.76)	24.84 ± 18.51 (2.92±73.32)	25.8 ± 21.58 (2.05–111.87)	0.804
Last follow-up (mm)	23.38 ± 17.36 (4.53–66.01)	20.91 ± 15.87 (0–72.76)	21.88 ± 16.37 (0–72.76)	0.577

**Table 5:** Radiological results in all patients and between groups

	H-A group	L-A group	Total	*P* value
Cobb angle				
Preoperation (°)	98.33 ± 22.88 (52.9–130.5)	87.83 ± 19.46 (47.1–126.2)	91.92 ± 21.3 (47.1–130.5)	0.064
Postoperation (°)	54.28 ± 18.05 (16.1–103.0)	52.96 ± 17.52 (23.2–90.6)	53.48 ± 17.59 (16.2–103)	0.013
Last follow-up (°)	48.4 ± 18.46 (5.1–73.5)	47.56 ± 16.06 (17.5–81.3)	47.89 ± 16.89 (5.1–81.3)	0.853
Cobb initial correction rate	45.07% ± 13.12% (10–69%)	39.6% ± 14.49% (17–66%)	41.73% ± 14.12% (10–69%)	0.025
Thoracic kyphosis				
Preoperation (°)	81.37 ± 31.92 (6–145.4)	54.14 ± 25.19 (4.4–102.8)	64.76 ± 30.8 (4.4–145.4)	0.001
Postoperation (°)	43.57 ± 19.55 (13–75.9)	31.53 ± 17.93 (5–83.3)	36.22 ± 19.34 (5–83.30)	0.018
Last follow-up (°)	50.1 ± 20.56 (16.5–81.5)	38 ± 17.25 (9.7–82.5)	42.72 ± 19.37 (9.7–82.5)	0.018
Lumbar lordosis				
Preoperation (°)	68.53 ± 20.14 (30–107)	55.22 ± 15.08 (25.1–91.1)	60.41 ± 18.27 (25.1–107)	0.005
Postoperation (°)	49.8 ± 14.33 (25.9–73.1)	45.21 ± 14.6 (10–72.2)	47 ± 14.54 (10–73.1)	0.266
Last follow-up (°)	49.8 ± 14.33 (25.9–73.1)	50.32 ± 11.67 (23.1–69.5)	47 ± 14.54 (10–73.1)	0.353
T1–S1 height				
Preoperation (mm)	228.82 ± 48.55 (160.41–318.06)	260.88 ± 46.06 (154.68–359.75)	248.38 ± 49.23 (154.68–359.75)	0.013
Postoperation (mm)	298.61 ± 37.75 (212.71–381.07)	321.79 ± 50.09 (198.96–425.52)	312.75 ± 46.74 (198.96–425.52)	0.063
Last follow-up (mm)	341.25 ± 47.46 (263.93–464.87)	367.08 ± 51.93 (254.93–465.51)	357.01 ± 51.41 (254.93–465.51)	0.059
T1–S1 initial increasing rate	33.62% ± 19.74% (6–79%)	24,25% ± 11.66% (10–55%)	27.9% ± 15.84% (6%–79%)	0.025
T1–T12 height				
Preoperation (mm)	128.26 ± 31.54 (68.1–190.48)	153.34 ± 35.96 (69.3–224.6)	143.56 ± 36.19 (68.1–224.6)	0.008
Postoperation (mm)	179.68 ± 28.53 (106.24–237.91)	189.85 ± 38.83 (87.88–261.91)	185.88 ± 35.26 (87.88–261.91)	0.284
Last follow-up (mm)	204.82 ± 33.35 (144.8–259.06)	216.88 ± 39.15 (123.35–292.06)	212.18 ± 37.14 (123.35–292.06)	0.227
T1–T12 initial increasing rate	45.37% ± 32.12% (9–159%)	25.49% ± 15.42% (3–84%)	33.24% ± 25.11% (3–159%)	0.002

## Complications

Overall, 78 complications (33 in the H-A group and 45 in the L-A group) were noted in 37 patients, and 60 (20 in the H-A group and 40 in the L-A group) were implant-related complications in 35 patients. The mean number of complications per patient was 1.43 (0–6) in the H-A group and 1.25 (0–5) in the L-A group. The mean number of implant-related complications per patient was 0.87 (0–3) in the H-A group and 1.11 (0–4) in the L-A group. The overall complication rate and implant-related complication did not differ significantly between both groups (*P* > 0.05). No significant differences were observed in implant dislodgement, rod fracture, proximal junctional kyphosis, and implant loosening between both groups (*P* > 0.05). Surgical site infection and neurological complications were similar between both groups (*P* > 0.05). Complications in all patient groups are shown in Table 6.

**Table 6:** Complications in all patients and between groups

	H-A group	L-A group	Total	*P* value
Total no. of complications	33/89	45/145	78/234	0.341
No. of patients with at least one complication	14	23	37	0.815
No. of patients with at least one implant-related complication	12	23	35	0.372
No. of complications per patient	1.43	1.25	1.32	
No. of implant-related complications per patient	0.87	1.11	1.02	
Complication rate per surgical procedure (%)	37.1	31	33.3	
Implant-related complication rate per surgical procedure (%)	22.5	27.6	25.6	
Implant-related complications	20/89	40/145	60/234	0.384
Implant dislodgement	5/89	5/145	10/234	0.426
Implant loosen	3/89	11/145	14/234	0.3
Rod fracture	3/89	14/145	17/234	0.072
Proximal junctional kyphosis	9/89	10/145	19/234	0.382
No. of patients with more than two complications	6	14	20	0.311
Neurological complication (no.)	2/89	1/145	3/234	0.667
Surgical site infection (no.)	4/89	1/145	5/234	0.051
Superficial	2/89	0/145	2/234	0.144
Deep	2/89	1/145	3/234	0.667
Other complications	7/89	3/145	10/234	0.033
Pulmonary problems	5/89	1/145	6/234	0.059
Gastrointestinal	1/89	2/145	3/234	1
Dural tear	1/89	0/145	1/234	0.38

## Discussion

Here, no significant difference was noted in the causes of scoliosis among patients at different altitudes (*P* = 0.291). The average age of patients in the H-A group was higher than that in the L-A group, although this difference was not significant (*P* = 0.064). However, the height and weight of the patients in the H-A group were significantly lower than that in the L-A group (*P* < 0.05), and the BMI of those in the H-A group was significantly lower than that of those in the L-A group (*P* = 0.036). Moreover, T1–S1 height and T1–T12 height in the L-A group were significantly higher than those in the H-A group preoperatively. The Cobb angle was more severe in the H-A group than that in the L-A group preoperatively, although this difference was not significant (*P* = 0.064). TK and LL in the H-A group were significantly larger than those in the L-A group (*P* = 0.01 and *P* = 0.05, respectively). The height, weight, BMI, T1–12, and T1–S1 of patients with EOS living in high-altitude areas were lower than those of patients in low altitude areas, which may be related to poor nutritional status and long-term hypoxic environment of children in high-altitude areas. Severe spinal deformities were more common in children in high-altitude areas, and also associated with small thoracic volume. The results showed that 73.9% of the patients in the high-altitude group were treated with dual rods, while only 16.7% of the patients in the low-altitude group were treated with dual rods. No significant difference was noted in the complications between both groups. Patients with scoliosis in high-altitude areas have been given increasing attention and treatment has been focused on them, which may be related to the improvement of concept and technology, and the progress of internal fixation technology. Related internal and medical complications were more common in patients with larger TK; if the TK exceeds the normal value, the rods and foundation anchors may bear more pressure and aggravate the failure of fixation [14].

Here, TK in the H-A group was significantly higher than that in the L-A group. TK is an important parameter to evaluate the severity of scoliosis. The increase or decrease of TK will affect the curative effect and complications of growing rod surgery for EOS. In a previous study, a surprisingly strong correlation between TK and Cobb has been reported [15]. This may be related to hypoxia, poor economic conditions, and malnutrition in children living in high-altitude areas, which is similar to the results of another previous study reporting that the nutritional status of Tibetan children is poor, and their incidence of malnutrition and dysplasia is high, which is related to high altitude [13]. The risk of stunting is 2–6 times higher in children living over 3500 m than those living over 3000 m. Moreover, the effect of high altitude on growth retardation persists in both young and older children [16]. Long-term malnutrition causes irreversible neurodevelopmental delays, leading to increased morbidity and mortality [17]. In particular, over one-third of children have stunted growth and are underweight, which are related to altitude, and the prevalence of stunting and underweight increases with altitude, increasing the dose-response relationship. Although socioeconomic factors play an increasingly important role in the growth of Tibetan children, altitude effects must be considered [18]. Altitude significantly increases the risk of neurodevelopmental problems in the first 2 years after birth. Moreover, it may significantly increase the risk of neurodevelopmental problems as high altitude increases the risk of neurodevelopmental diseases in women by 3 times, and in men by 1.9 times. This may be because firstly, oxygen levels are low and uterine blood flow is reduced in high-altitude areas, resulting in decreased oxygen flow to the fetus, which may cause permanent nerve damage; secondly, high altitude may also have adverse effects on maternal health, such as an increased risk of preeclampsia and pregnancy-induced hypertension, thereby affecting fetal development [16]. The incidence of rib deformity in children with congenital scoliosis at high altitudes is high, and their degree of rib deformity is severe [13].

Children with EOS in high-altitude areas have poor nutritional status, shorter height, lighter weight, severe spinal deformity, which may be combined with spinal cord or vascular malformation, thoracic deformity, small thoracic volume, worse cardiopulmonary function, and commonly have restrictive ventilation dysfunction, and even respiratory failure. Therefore, more comprehensive preoperative evaluation is needed because Tibetan children in high-altitude areas have language communication difficulties. Thus, health education should be carried out for these children, and preoperative nutrition and balloon blowing exercise are needed. Preoperative traction treatment and intraoperative nerve electrophysiological monitoring should be used. Dual rods implanted subcutaneously should be used as much as possible to maintain spinal growth, especially for children with EOS in high-altitude areas. However, for patients with poor nutritional status and larger Cobb angle, inserting dual growing rods may be difficult, and the anchoring strength of the lower and upper internal fixation should be increased as much as possible to ensure the role of growing rods in spinal correction. During the operation, the integrity of the capsule and the posterior ligament of the adjacent segment of the fixed vertebrae should be protected to prevent the failure of internal fixation. The first operation and each distraction operation are carried out under spinal cord monitoring, and the intraoperative blood pressure of patients is always a concern. Once the spinal cord monitoring signal appears abnormal during the operation, the factors that may lead to nerve injury should be investigated first, the distraction should be relaxed, the blood pressure changes should be checked, and hormonal treatment should be done. As patients in high-altitude areas are mostly Tibetan children and their language communication may not be smooth, X-ray taking cannot be done on time, so there may be difficulty in growing rod adjustment. Thus, more attention should be paid to the healthcare of patients in high-altitude areas. EOS is an extremely complex disease, and various pathogenic factors lead to several prognostic changes; if not promptly treated, scoliosis will gradually worsen, which may lead to shortening of the trunk, hindering of lung development, and early heart and lung failure [17]. Progressive scoliosis reduces chest compliance and shifts and rotates the contents of the chest cavity, resulting in asymmetry in lung size [4]. The treatment of severe spinal deformity in children is a huge challenge, the failure rate of non-surgical treatment is high, and spinal fusion correction can lead to a short trunk [5]. The core concept of the treatment of EOS is to maintain growth while controlling further aggravation of the deformity. TGR technology has achieved good clinical effects in the treatment of EOS [8, 11, 17]. A single rod can achieve a good clinical effect, although it cannot provide sufficient spinal support when the patient is active [6]. The dual growing rod technique can achieve better coronal correction rate and lengthening [6, 19]. Despite satisfactory deformity correction and skeletal maturity, permanently retaining the double growing rod can be considered [20].

Mahdavi [19] used double growing rods to treat 22 patients with EOS, in which the Cobb angle of the patients was changed from 52 ± 24° preoperatively to 38 ± 19° at the last follow-up, and the TK angle was changed from 78 ± 22° preoperatively to 60 ± 17° at the last follow-up, although the incidence of implant-related complications was high at 54.5%. In our previous study, the growing rod treatment of EOS, in which main indicators of thoracic scoliosis and TK were significantly improved at the last follow-up compared with those recorded during the preoperative period [20]. Single growing rods can effectively improve the angle of scoliosis and maintain spinal growth, although they are not equivalent with the dual rods in preventing internal fixation breakage and maintaining the corrective angle [21]. Here, 30 patients (83.3%) were treated with a single rod, and 6 patients (26.1%) were treated with a dual rod (*P* < 0.05). Dual rod surgery has been mainly completed recently because the foundation has funded the treatment of children in high-altitude areas, with a total of 12 patients in the H-A group and 18 in the L-A group who underwent subcutaneous growing rod implantation, while the other patients were implanted with growing rods submuscularly (*P* = 0.871). A meta-analysis of single and dual rod treatment of EOS has showed that dual growing rod implantation can achieve better deformity correction and spinal growth. The incidence of complications related to internal fixation was lower in the in the single rod group than in the dual rod group [6]. Here, although there were differences in the application of single and dual rods in the two groups, both rod types achieved good therapeutic effects.

The incidence of IRC for spinal surgery was 0.19%, and the incidence of IRC for three-column correction surgery for spinal deformity was 40.2% [22]. TGR technology not only improves the longitudinal growth of the thoracic cavity but also increases the width of the thoracic cavity; thus, the volume of the thoracic cavity can be significantly increased during the treatment process. However, as the age and the number of extensions increase, the width of the thorax decreases, which is related to the stiffness and spontaneous fusion of the spine; however, the authors also pointed out that for patients with severe EOS, growing rod implantation cannot be postponed because multiple adjustment rod operations can cause an increase in the absolute value [3]. The incidence of surgical complications was relatively high at 58–79% in the treatment of EOS, and repeated anesthesia and surgical procedures are major problems [8, 11, 17]. The incidence of internal fixation-related complications was 54.2%, and the incidence of surgical site infection was 22.7% [19]. Bouthors [21] retrospectively analyzed 34 patients with single growing rods for EOS. Although single growing rods can achieve good results in deformity correction and maintenance of spinal growth, the IRCs are especially high. Hence, it is recommended that dual growing rods be used for patients over 8 years old as such rods can better prevent breakage and maintain the orthopedic effect for patients with satisfactory orthopedics after bone maturity. Additionally, dual growing rods can be retained instead of performing final fusion surgery [21].

Here, the overall incidence of complications in the H-A and L-A groups was 60.9% and 63.9%, respectively, with no significant difference (*P* = 0.815). The incidence of implant-related complications in the L-A group (63.9%) was higher than that in the H-A group (52.2%), although the difference was not significant (*P* = 0.372). 3D print can help to understand the severity of spinal deformity more clearly and facilitate preoperative communication between doctors and patients, 3D printing technology has a good prospect in spinal deformity surgery. In recent years, our institute has begun to use 3D printing for preoperative evaluation of surgical treatment of scoliosis [23]. Most children with EOS in the H-A group have been subsidized through spinal rescue action recently, and the progress of the treatment concept and the improvement of surgical techniques and internal fixation equipment all promote further reduction of implant-related complications.

Our study has several limitations. First, this was a retrospective study, and no randomization was performed between the H-A and L-A groups. Second, the number of patients was relatively small; thus, a larger number of patients and a longer follow-up period are needed. Third, the operative time of most patients in the L-A group was shorter than that in the H-A group, and the proportion of patients using a single rod or dual rod was very different between the groups, which may have affected the study results.

## Conclusions

The deformity of patients with EOS in high-altitude areas is more severe. The height, weight, and BMI of the H-A group were lower than those in the L-A group, and the differences were significant. The T1–S1 and T1–12 ranges in the H-A group were smaller than those in the L-A group. TK and LL in the H-A group were also greater than those in the L-A group. The PI, PT, and SS values of both groups were not significantly different. The use of growing rods achieved good therapeutic effects in both groups, although complications and internal fixation-related complications were frequent in both groups. Thus, children with EOS in high-altitude areas deserve more attention.

## Data Availability

The datasets used and analyzed during the current study are available from the corresponding author on reasonable request.

## Abbreviations

TGR: Traditional growing rod
H-A group: High-altitude group
L-A group: Low-altitude group
TK: Thoracic kyphosis
LL: Lumbar lordosis
PI: Pelvic incidence
SS: Sacral slope
PT: Pelvic tilt
EOS: Early-onset scoliosis
MCGRs: Magnetically controlled growing rods
VEPTR: Vertical expandable titanium rib prosthesis
PACS: Picture archiving and communication system
LIV: Lower instrumental vertebra
UIV: Upper instrumental vertebra
BMI: Body mass index
